# Post-marketing safety of ensifentrine in COPD: A retrospective pharmacovigilance study using disproportionality analysis of FAERS/AEMS reports

**DOI:** 10.1097/MD.0000000000050743

**Published:** 2026-09-25

**Authors:** Fatma Özge Yağbasan

**Affiliations:** a Department of Medical Services and Techniques, Vocational High School of Health Services, Giresun University, Giresun, Türkiye.

**Keywords:** AEMS, COPD, disproportionality, ensifentrine, FAERS, MedDRA, pharmacovigilance

## Abstract

Ensifentrine is a newly approved inhaled dual phosphodiesterase 3 and 4 inhibitor for the maintenance treatment of chronic obstructive pulmonary disease. Because post-marketing safety experience is still limited, this study evaluated adverse event reporting patterns for ensifentrine. Quarterly US Food and Drug Administration (FDA) Adverse Event Reporting System/Adverse Event Monitoring System files from 2024Q3 to 2026Q1 were analyzed. Duplicate reports were removed using Case Identification Number (CASEID), FDA receipt date (FDA_DT), and Primary Identification Number (PRIMARYID), and the primary analysis was restricted to reports in which ensifentrine was recorded as the primary suspect drug. Preferred Terms (PTs) were mapped to Medical Dictionary for Regulatory Activities primary System Organ Class categories, and report-level disproportionality was assessed using reporting odds ratio, proportional reporting ratio, an information component approximation, and an observed-to-expected approximation. After deduplication, 2,831,030 records were reduced to 2,500,712 unique reports. Ensifentrine was identified in 1137 reports across all drug roles and in 823 primary-suspect reports, which included 1817 PT records. At the System Organ Class level, Respiratory, thoracic and mediastinal disorders was the only category meeting all predefined robust signal criteria. At the PT level, respiratory, cardiovascular, psychiatric/neuropsychiatric, and product quality- or medication-use-related terms were notable. Time-to-onset could be calculated for 139 reports, with a median of 12 days. Respiratory reporting patterns predominated in early post-marketing reports for ensifentrine. These findings should be interpreted as hypothesis-generating pharmacovigilance signals rather than estimates of incidence or causality.

## 1. Introduction

Chronic obstructive pulmonary disease (COPD) is a common chronic disease in older adults, characterized by dyspnea, cough, excessive mucus production, and exacerbations, and it is usually associated with largely irreversible and progressive airflow limitation.^[1,2]^ Current treatment options, including inhaled corticosteroids (ICSs) and oral phosphodiesterase-4 (PDE4) inhibitors, remain largely symptomatic and may be limited by adverse effects, highlighting the need for new therapeutic approaches.^[3,4]^ In this context, spontaneous reports concerning newly approved COPD therapies require careful interpretation because reported events may reflect potential adverse reactions, manifestations of the underlying disease, treatment indications, common comorbidities, or other reporting factors.

Ensifentrine is an inhaled, selective dual phosphodiesterase 3 (PDE3) and PDE4 inhibitor with bronchodilatory and anti-inflammatory properties.^[1,2]^ In the phase III ENHANCE-1 and ENHANCE-2 trials, nebulized ensifentrine 3 mg administered twice daily showed favorable effects on lung function and patient-reported outcomes. Based on these findings, ensifentrine has been evaluated as a novel maintenance treatment option for adult patients with COPD, and inhaled ensifentrine suspension was approved in the United States in June 2024 for the maintenance treatment of COPD in adults.^[2,5–7]^

The US Food and Drug Administration Adverse Event Reporting System (FAERS) is one of the largest pharmacovigilance databases used to evaluate drug safety in real-world settings. It is widely used to detect rare or serious adverse events and to identify potential post-marketing safety signals.^[8]^ Spontaneous reporting systems such as FAERS/Adverse Event Monitoring System (AEMS) support post-marketing signal detection and pharmacovigilance surveillance.^[9–11]^ However, the presence of a report in FAERS/AEMS does not establish that the drug caused the event, nor does it allow estimation of event incidence.^[12,13]^

Therefore, this study was designed to characterize early post-marketing adverse event reports submitted for ensifentrine, to evaluate report-level disproportionality patterns at the Preferred Term (PT) and Medical Dictionary for Regulatory Activities (MedDRA) primary System Organ Class (SOC) levels, and to clearly distinguish patient-level adverse events from disease- or indication-related terms, product quality reports, and medication-use or effectiveness-related reports. The aim was to provide an early, exploratory assessment of the potential safety reporting profile of ensifentrine using the FAERS/AEMS database.

## 2. Materials and methods

### 2.1. Data source

This disproportionality analysis was based on quarterly American Standard Code for Information Interchange files from FAERS/AEMS. Given that inhaled ensifentrine suspension was approved for the maintenance treatment of COPD in June 2024, this study analyzed FAERS/AEMS quarterly data files from 2024Q3 to 2026Q1. The quarterly American Standard Code for Information Interchange extract files used in the analysis were downloaded from the US Food and Drug Administration (FDA) website on June 6, 2026.

The FDA states that these files are raw, noncumulative extracts from the FAERS/AEMS database for the corresponding reporting periods.^[14]^ In FAERS/AEMS, adverse event and medication error terms are coded using MedDRA terminology.^[15]^ This study used exclusively publicly available and deidentified FAERS/AEMS data and involved no direct interaction with human participants. Therefore, institutional ethics committee or institutional review board approval and individual informed consent were not required.

### 2.2. Data processing

Duplicate reports in the demographic and administrative information table (DEMO) were removed using CASEID, FDA receipt date (FDA_DT), and Primary Identification Number (PRIMARYID), following the FDA-recommended approach. For reports with the same CASEID, the record with the most recent FDA_DT was retained; when multiple records had the same CASEID and FDA_DT, the report with the highest PRIMARYID was kept. The retained PRIMARYID values were then used to filter the drug information (DRUG), adverse reaction (REAC), patient outcome (OUTC), drug therapy (THER), drug indication (INDI), and report source (RPSR) tables.

The target drug was identified in the DRUG table by searching both the drug name field (DRUGNAME) and product active ingredient field (PROD_AI) for the generic name “ENSIFENTRINE” and the brand name “OHTUVAYRE.” Before matching, all entries were converted to uppercase and leading and trailing whitespace was removed. Substring matching was performed using the expression “ENSIFENTRINE|OHTUVAYRE,” which also captured entries containing additional formulation, strength, or product information. Thus, capitalization and whitespace variations were accommodated, whereas no additional misspelled forms or fuzzy-matching procedures were used. The primary analysis was subsequently restricted to unique reports in which the matched drug was coded as the primary suspect (ROLE_COD = “PS”).

For disproportionality analyses, the unit of analysis was defined as the unique report rather than the PT record. For each PT or SOC, a report-level 2 × 2 contingency table was constructed. If the same PT or SOC appeared more than once within a single report, that report was counted only once for the corresponding PT or SOC (Table 1). Reporting complexity was summarized as the number of unique PTs per report. A report was classified as containing multiple suspect drugs when more than 1 unique drug was coded as a primary or secondary suspect (ROLE_COD = “PS” or “SS”).

**Table 1:** Report-level 2 × 2 contingency table for PT- and SOC-level disproportionality analyses.

	Event/SOC reported	Event/SOC not reported
Ensifentrine PS reports	***a***	***b***
Non-ensifentrine reports in the reference set	***c***	***d***

Counts represent unique reports, not PT records. For each PT or SOC, *a* denotes ensifentrine PS reports with the event/SOC; *b* denotes ensifentrine PS reports without the event/SOC; *c* denotes non-ensifentrine reports in the reference set with the event/SOC; and *d* denotes non-ensifentrine reports in the reference set without the event/SOC. The reference set consisted of deduplicated FAERS/AEMS reports from 2024Q3 to 2026Q1 with at least one REAC record, excluding target ensifentrine PS reports.

AEMS = Adverse Event Monitoring System, FAERS = FDA Adverse Event Reporting System, PS = primary suspect, PT = Preferred Term, Q = quarter, REAC = adverse reaction, SOC = System Organ Class.

The PTs in the REAC table were mapped to their primary SOCs using the English MedDRA version 29.0 (mdhier.asc; MedDRA Maintenance and Support Services Organization [MSSO]) hierarchy file. Because a single PT may be linked to >1 SOC in MedDRA, the primary SOC assignment was used to avoid double counting.

### 2.3. Statistical analysis

Reporting patterns at the PT and SOC levels for ensifentrine were evaluated using disproportionality analysis. Report-level reporting odds ratio (ROR), proportional reporting ratio (PRR), an information component (IC) approximation, and a supportive observed-to-expected (OE) approximation were calculated (Table 2). To avoid infinite estimates in cells with small counts, the Haldane–Anscombe 0.5 continuity correction was applied.^[16–19]^

**Table 2:** Summary of disproportionality metrics used in this analysis.

Method	Formula/unit	Signal criterion
ROR	ROR = (*a* × *d*)/(*b* × *c*); counts are unique reports	ROR025 > 1 and n ≥ 2
PRR	PRR = [*a*/(*a* + *b*)]/[*c*/(*c* + *d*)]	PRR ≥ 2, χ² ≥ 4 and n ≥ 3
IC approximation	IC = log2[*a* × N/((*a* + *c*)(*a* + *b*))]	IC025 > 0
OE approximation	OE = *a* × N/[(*a* + *c*)(*a* + *b*)]	OE025 > 2

In the signal criteria, n = *a*, that is, the number of unique reports containing both ensifentrine and the event/SOC. N = *a* + *b* + *c* + *d*. A 0.5 continuity correction was applied. OE and OE025 represent non-shrunken OE approximations and should not be interpreted as EBGM or EB05 estimates derived from a formal MGPS model.

EB05 = empirical Bayes 5th percentile, EBGM = empirical Bayes geometric mean, IC = information component, IC025 = lower 95% confidence bound of the information component, MGPS = multi-item gamma Poisson shrinker, OE = observed-to-expected, OE025 = lower 95% confidence bound of the observed-to-expected ratio, PRR = proportional reporting ratio, ROR = reporting odds ratio, ROR025 = lower 95% confidence bound of the reporting odds ratio, SOC = System Organ Class.

The signal criteria were defined as follows: ROR025 > 1 with n ≥ 2 for ROR; PRR ≥ 2, χ^2^ ≥ 4, and n ≥ 3 for PRR; IC025 > 0 for the IC approximation; and OE025 > 2 for the OE approximation.

A crude OE ratio and its approximate lower 95% confidence bound (OE025) were calculated as supportive descriptive measures. The OE025 > 2 threshold was prespecified as a conservative supportive filter, requiring the lower bound of the approximate 95% interval for the crude OE ratio to remain above a 2-fold elevation. The 2-fold cutoff was selected pragmatically to increase the stringency of signal screening and to align with the magnitude component of the commonly used PRR ≥ 2 criterion.^[12,18,19]^ This threshold was not considered a validated Bayesian criterion. Because the OE measure used in this study did not incorporate empirical Bayesian shrinkage, OE025 was not interpreted as empirical Bayes 5th percentile, empirical Bayes geometric mean (EBGM), multi-item gamma Poisson shrinker (MGPS), or formal Bayesian confidence propagation neural network (BCPNN) output.

Here, n refers to cell *a* in the 2 × 2 table, that is, the number of unique reports containing both ensifentrine and the relevant event or SOC. The OE metric was reported as a supportive OE approach and was not interpreted as a formal MGPS/EBGM model. Formal Bayesian shrinkage methods, including EBGM/MGPS and full BCPNN, were not applied in this study. The analysis was designed as an early report-level pharmacovigilance assessment using transparent and reproducible disproportionality measures. Therefore, the IC and OE metrics were used as supportive approximations and were not interpreted as formal Bayesian shrinkage estimates.^[19]^

A **robust reporting signal** was defined as a PT or SOC that met all predefined ROR, PRR, IC, and OE criteria. In the clinical interpretation of PT-level findings, terms that could be confounded by the underlying disease or indication, as well as terms related to product quality, preparation, effectiveness, or medication use, were evaluated separately from clinical adverse events.

Time-to-onset was calculated as the number of days between the event date and the therapy start date in reports with valid information for both fields. Reports with missing, negative, or implausibly long values were excluded; values longer than 650 days were considered likely date-entry errors for a newly approved drug. Time-to-onset findings were presented descriptively only. All data cleaning, mapping, and statistical analyses were performed using R version 4.6.0 (R Foundation for Statistical Computing) through the RStudio IDE (Posit Software, PBC), with the data.table, dplyr, stringr, lubridate, and readr packages.

### 2.4. Sensitivity analyses

Five sensitivity analyses were conducted at both the PT and primary SOC levels using the same report-level measures, continuity correction, and signal thresholds as in the primary analysis. These analyses included ensifentrine reports from all drug-role codes; reports listing exactly 1 unique suspect drug coded as PS or SS; target and reference reports containing a COPD indication; reports submitted by healthcare professionals, defined by reporter occupation codes physician (MD), pharmacist (PH), other health professional, or health professional; and exclusion of reports containing a PT assigned to the MedDRA primary SOC Product issues. The same eligibility criterion was applied to the ensifentrine and reference populations in each analysis.

Stability was evaluated using the number of robust PT and SOC signals, overlap with the primary-analysis signals, signal-retention percentage, Jaccard index, and Spearman correlation of log-transformed ROR estimates. Detailed definitions and results are provided in Tables S1, S2 and S3, Supplemental Digital Content 1.

## 3. Results

### 3.1. Report selection and baseline characteristics

During the 2024Q3 to 2026Q1 study period, the DEMO table contained 2,831,030 records. After deduplication, 2,500,712 unique reports remained. Ensifentrine was identified in 1137 reports across all drug roles and in 823 reports in the primary suspect (PS)-only analysis. These 823 reports included 1817 PT records (Fig. 1). At the report level, the median number of unique PTs was 2 (interquartile range [IQR], 1–3; range, 1–14). Eighteen reports (2.2%) contained >1 unique PS or SS drug, whereas 805 reports (97.8%) contained a single suspect drug. Sex information was available in 612 reports, including 329 females and 283 males. Age was reported in 219 cases, with a median age of 74 years (IQR, 67–79). All reports originated from the United States. In the indication field, “Product used for unknown indication” was the most frequently entered term, whereas COPD was the predominant clinical disease term. “Product used for unknown indication” was recorded in 605 of the 815 reports containing at least 1 INDI entry (74.2%). Because all drug–indication records within target reports were summarized at the report level and indication categories were not mutually exclusive, an unknown-indication entry could coexist with a COPD indication or could relate to another drug listed in the same report. Accordingly, this percentage should not be interpreted as indicating that the clinical indication for ensifentrine was unknown in 74.2% of cases. Regarding outcomes, 265 reports were classified as serious; death was reported in 10 cases, corresponding to 1.2% of all PS-only reports and 3.8% of serious reports. Among the 10 reports coded with death as an outcome, 5 contained at least 1 respiratory PT, 2 contained an infection PT (pneumonia in both), 1 contained a cardiac PT (atrial fibrillation), and none contained an explicit COPD exacerbation PT. The categories were not mutually exclusive: one report contained both acute respiratory failure and pneumonia, while another contained dyspnea and atrial fibrillation. Three reports listed completed suicide as the sole PT, and 1 additional report included suicidal ideation and depressed mood together with respiratory PTs. One report listed metastatic lung cancer. These PTs were co-reported with the death outcome and were not adjudicated causes of death (Table S4, Supplemental Digital Content 2; Table 3).

**Table 3:** Characteristics of reported adverse event cases associated with ensifentrine.

Characteristic	Available n	Case n	Within available/subgroup (%)	% of all PS reports
Ensifentrine reported AE cases, PS-only	823	823	100.00	100.00
Reported adverse event PT records	1817	1817	–	–
PTs/report, median (IQR)	823	2 (1–3)	–	–
>1 suspect drug, n (%)	823	18	2.19	2.19
Sex, n (%)	612	–	74.36	–
Female	–	329	53.76	39.98
Male	–	283	46.24	34.39
Age (yr), n (%)	219	–	26.61	–
<18	–	0	0.00	0.00
18–65	–	41	18.72	4.98
>65	–	178	81.28	21.63
Median (IQR)	–	74 (67–79)	–	–
Weight (kg), n (%)	89	–	10.81	–
<80	–	62	69.66	7.53
80–100	–	16	17.98	1.94
>100	–	11	12.36	1.34
Median (IQR)	–	70.2 (59.9–84.0)	–	–
Reported country, n (%)	823	–	100.00	–
US	–	823	100.00	100.00
Indications, n (%)	815	–	99.03	–
Product used for unknown indication	–	605	74.23	73.51
Chronic obstructive pulmonary disease	–	391	47.98	47.51
Emphysema	–	28	3.44	3.40
Dyspnea	–	6	0.74	0.73
Asthma	–	5	0.61	0.61
Concomitant drugs, n (%)	233	–	28.31	–
Trelegy Ellipta	–	62	26.61	7.53
Albuterol sulfate	–	61	26.18	7.41
Albuterol	–	53	22.75	6.44
Breztri	–	43	18.45	5.22
Budesonide	–	27	11.59	3.28
Outcomes, n (%)	823	–	100.00%	–
Nonserious outcome	–	558	67.80	67.80
Serious outcome	–	265	32.20	32.20
Death	–	10	3.77% of serious; 1.22% of all	1.22
Life-threatening	–	12	4.53% of serious; 1.46% of all	1.46
Hospitalization	–	122	46.04% of serious; 14.82% of all	14.82
Disability	–	0	0.00	0.00
Other serious outcomes	–	165	62.26% of serious; 20.05% of all	20.05
Time-to-onset (d)	139	–	16.89	–
Median (IQR)	–	12 (0–57)	–	–
0–7 d	–	62	44.60	7.53
8–30 d	–	29	20.86	3.52
31–90 d	–	20	14.39	2.43
>90 d	–	28	20.14	3.40
Reporters, n (%)	559	–	67.92	–
Health professional	–	330	59.03	40.10
Consumer	–	229	40.97	27.82
Reporting period, n (%)	823	–	100.00	–
2024Q3	–	1	0.12	0.12
2024Q4	–	12	1.46	1.46
2025Q1	–	106	12.88	12.88
2025Q2	–	110	13.37	13.37
2025Q3	–	144	17.50	17.50
2025Q4	–	178	21.63	21.63
2026Q1	–	272	33.05	33.05

“Available n” represents the number of reports with available data for the relevant variable. Percentages in the “Within available/subgroup” column were calculated using the following variable-specific denominators: sex, n = 612; age, n = 219; weight, n = 89; reported country, n = 823; indication, n = 815; reports containing at least one concomitant drug, n = 233; outcomes, n = 823; evaluable time-to-onset, n = 139; reporter type, n = 559; and reporting period, n = 823. Percentages in the “% of all PS reports” column were calculated using all 823 primary-suspect reports. Percentages for individual serious outcomes were calculated among the 265 serious reports, with their corresponding percentages among all 823 PS reports also presented. Missing data were excluded from variable-specific denominators. Indication, concomitant drug, and serious-outcome categories were not mutually exclusive and may therefore sum to more than 100%. PT counts were based on unique PTs after within-report deduplication, and multiple suspect drugs denote more than one unique drug coded as PS or SS. “Product used for unknown indication” could relate to another drug within the same report and does not necessarily indicate that the indication for ensifentrine was unknown.

AE = adverse event, IQR = interquartile range, PS = primary suspect, PT = Preferred Term, Q = quarter, SS = secondary suspect, US = United States.

**Figure 1.**
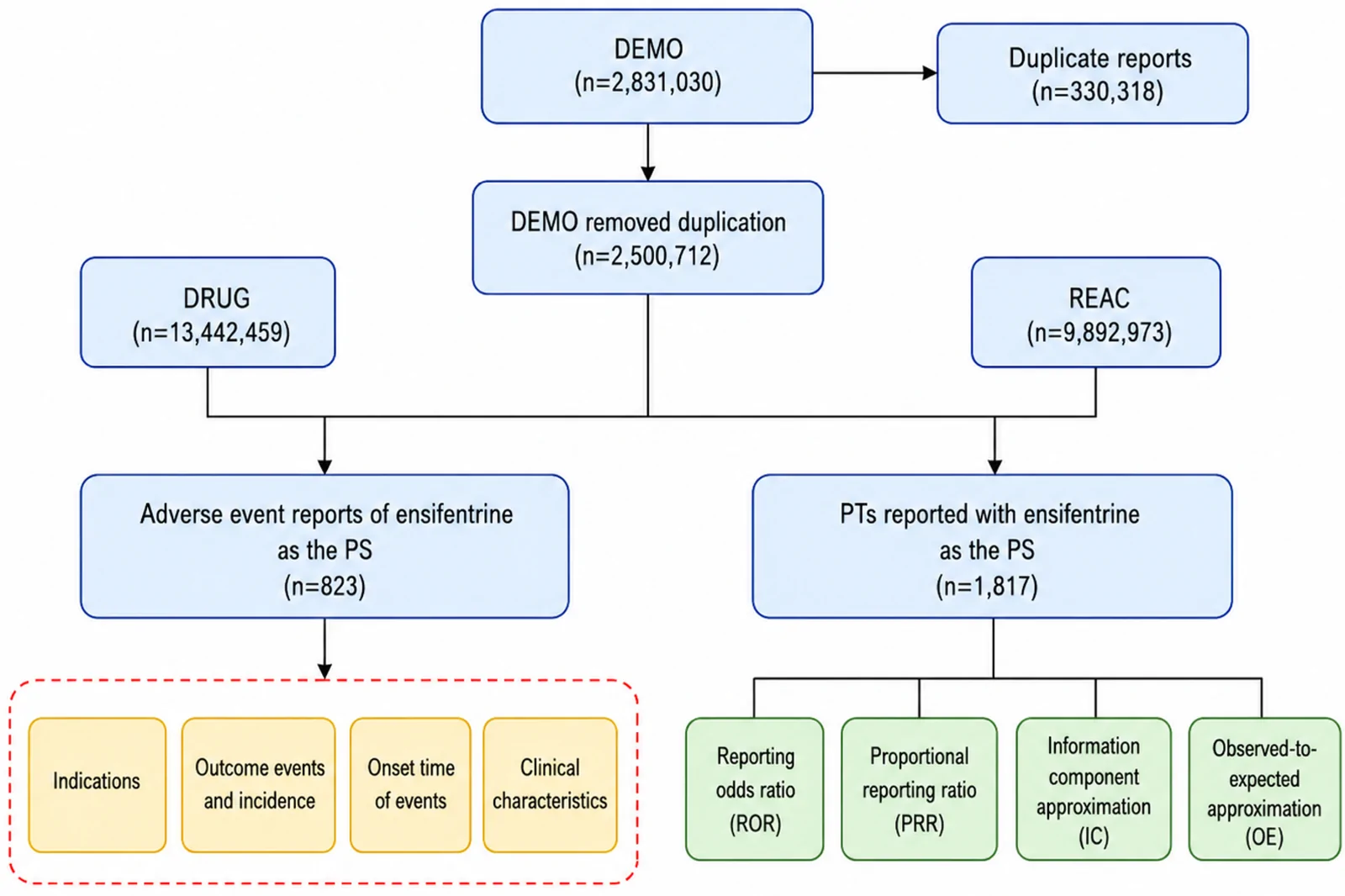
Flow diagram of selecting ensifentrine-related reported adverse events from FAERS/AEMS. AEMS = Adverse Event Monitoring System, DEMO = demographic and administrative information table, DRUG = drug information table, FAERS = FDA Adverse Event Reporting System, PS = primary suspect, PT = Preferred Term, REAC = adverse reaction table.

### 3.2. SOC-level disproportionality signals

Using MedDRA primary SOC mapping, 23 SOC categories were evaluated (Table 4). Among ensifentrine PS reports, the most frequently represented SOC was Respiratory, thoracic and mediastinal disorders (n = 315). This SOC was also the only robust SOC signal that met all 4 predefined signal criteria (ROR = 5.87, ROR025 = 5.10; PRR = 4.00; IC025 = 1.81; OE025 = 3.51). Although Psychiatric disorders had an ROR of 2.02, it did not fully meet the supportive PRR and OE criteria and was therefore not classified as a robust SOC signal. **Cardiac disorders** was reported in 48 cases but did not show a robust SOC-level signal.

**Table 4:** Signal values at the MedDRA primary SOC level.

SOC	Case number	ROR (95% CI)	PRR	χ²	IC025	OE025	Robust signal
Respiratory, thoracic and mediastinal disorders	315	5.87 (5.10–6.75)	4.00	785.08	1.81	3.51	Yes
General disorders and administration site conditions	211	0.60 (0.51–0.70)	0.70	43.24	−0.73	0.60	No
Psychiatric disorders	132	2.02 (1.68–2.44)	1.86	57.55	0.63	1.55	No
Gastrointestinal disorders	105	0.80 (0.66–0.99)	0.83	4.39	−0.56	0.68	No
Injury, poisoning and procedural complications	101	0.30 (0.25–0.37)	0.39	143.60	−1.66	0.32	No
Nervous system disorders	91	0.69 (0.55–0.86)	0.72	11.41	−0.78	0.58	No
Musculoskeletal and connective tissue disorders	81	1.07 (0.85–1.35)	1.07	0.36	−0.24	0.85	No
Investigations	70	0.75 (0.59–0.95)	0.77	5.53	−0.73	0.60	No
Infections and infestations	61	0.58 (0.44–0.75)	0.61	17.85	−1.09	0.47	No
Product issues	54	1.12 (0.85–1.47)	1.11	0.63	−0.25	0.84	No
Cardiac disorders	48	1.29 (0.97–1.73)	1.28	3.02	−0.07	0.95	No
Vascular disorders	37	0.87 (0.63–1.21)	0.88	0.70	−0.66	0.63	No
Skin and subcutaneous tissue disorders	34	0.32 (0.23–0.45)	0.35	48.12	−2.02	0.25	No
Renal and urinary disorders	20	0.63 (0.41–0.98)	0.64	4.19	−1.27	0.42	No
Surgical and medical procedures	20	0.49 (0.32–0.77)	0.51	10.31	−1.61	0.33	No
Eye disorders	16	0.40 (0.25–0.66)	0.42	14.25	−1.97	0.26	No
Immune system disorders	11	0.40 (0.22–0.71)	0.40	10.46	−2.15	0.23	No
Metabolism and nutrition disorders	11	0.26 (0.15–0.47)	0.27	23.77	−2.72	0.15	No
Social circumstances	5	0.44 (0.19–1.01)	0.44	3.96	−2.39	0.19	No
Neoplasms benign, malignant and unspecified (incl cysts and polyps)	3	0.09 (0.03–0.25)	0.09	33.18	−4.97	0.03	No
Ear and labyrinth disorders	2	0.27 (0.08–0.94)	0.27	4.84	−3.66	0.08	No
Blood and lymphatic system disorders	2	0.07 (0.02–0.23)	0.07	32.97	−5.65	0.02	No
Hepatobiliary disorders	1	0.07 (0.01–0.35)	0.07	18.61	−6.12	0.01	No

A robust signal indicates that all prespecified disproportionality signal criteria were simultaneously met.

CI = confidence interval, IC025 = lower 95% confidence bound of the information component, MedDRA = Medical Dictionary for Regulatory Activities, OE025 = lower 95% confidence bound of the observed-to-expected ratio, PRR = proportional reporting ratio, ROR = reporting odds ratio, SOC = System Organ Class.

### 3.3. PT-level signals and interpretation categories

A total of 46 robust reporting signals were identified at the PT level. For the main clinical interpretation, terms that could represent patient-level adverse events were distinguished from terms that could be confounded by the underlying disease or indication, as well as from product quality- or medication-use-related terms (Tables 5 and 6). Terms such as dyspnea and COPD were flagged as reporting patterns that may overlap with COPD symptoms or the treatment indication. In contrast, terms such as product color issue, liquid product physical issue, and product preparation issue were presented as product quality- or preparation-related reports rather than evidence of clinical adverse events.^[20]^

**Table 5:** Selected clinical PT-level disproportionality signals for ensifentrine PS-only reports, grouped by clinical domain and interpretive relevance.

PT	n	ROR	ROR025	PRR	χ²	IC025	OE025	Interpretation flag
Bronchospasm Paradoxical	12	1149.45	598.12	1132.03	10,287.17	8.78	439.71	Respiratory
Bronchospasm	20	49.28	31.68	48.08	930.85	4.93	30.45	Respiratory
Haemoptysis	9	10.25	5.40	10.15	78.16	2.41	5.33	Respiratory
Throat Tightness	5	5.86	2.53	5.83	22.00	1.33	2.52	Respiratory
Productive Cough	14	5.48	3.26	5.40	52.05	1.68	3.21	Respiratory
Sputum Increased	8	74.33	37.51	73.57	594.17	5.18	36.27	Respiratory
Sputum Discoloured	6	13.27	6.12	13.18	72.87	2.60	6.06	Respiratory
Hypertensive Crisis	7	23.06	11.20	22.86	155.64	3.46	11.03	Cardiovascular
Atrial Fibrillation	20	6.13	3.95	6.00	85.63	1.95	3.86	Cardiovascular
Chest Pain	23	4.05	2.69	3.96	52.39	1.39	2.63	Cardiovascular
Chest Discomfort	27	6.49	4.43	6.30	123.15	2.11	4.30	Cardiovascular
Blood Pressure Increased	21	3.58	2.33	3.51	38.88	1.19	2.29	Cardiovascular
Suicidal Ideation	22	7.55	4.96	7.37	123.96	2.27	4.83	Psychiatric
Depression	28	4.56	3.14	4.43	76.29	1.61	3.05	Psychiatric
Depressed Mood	10	4.56	2.48	4.52	28.80	1.30	2.45	Psychiatric
Hallucination	16	4.93	3.02	4.85	50.51	1.57	2.97	Psychiatric
Abnormal Dreams	7	12.06	5.87	11.96	75.09	2.54	5.80	Psychiatric
Feeling Jittery	6	12.24	5.65	12.15	66.31	2.48	5.59	Clinical/other
Back Pain	63	8.00	6.19	7.46	358.32	2.53	5.77	Clinical
Dyspnoea	174	10.04	8.49	8.13	1116.68	2.78	6.88	Disease-confounded
Chronic Obstructive Pulmonary Disease	21	11.49	7.48	11.22	199.79	2.86	7.28	Disease-confounded

n denotes unique target PS reports mentioning the PT. PTs are grouped by clinical domain and ordered according to clinical and interpretive relevance rather than the magnitude of the disproportionality estimates. Disproportionality estimates based on small report counts may be unstable and should not be interpreted as measures of incidence or clinical risk magnitude.

IC025 = lower 95% confidence bound of the information component, OE025 = lower 95% confidence bound of the observed-to-expected ratio, PRR = proportional reporting ratio, PS = primary suspect, PT = Preferred Term, ROR = reporting odds ratio, ROR025 = lower 95% confidence bound of the reporting odds ratio.

**Table 6:** Medication-use, effectiveness, preparation, and product-quality PTs, grouped by interpretive relevance and separated from clinical adverse event interpretation.

PT	n	ROR	ROR025	PRR	χ²	IC025	OE025	Group
Intentional Underdose	10	52.67	28.51	52.01	516.57	4.79	27.69	Medication use
Absence Of Immediate Treatment Response	5	179.65	75.82	178.46	916.68	6.15	71.25	Effectiveness/use
Drug Effect Less Than Expected	14	7.51	4.46	7.39	80.17	2.13	4.39	Effectiveness/use
Product Preparation Issue	12	33.81	19.28	33.31	387.67	4.23	18.80	Product quality/preparation
Product Solubility Abnormal	10	47.90	25.94	47.30	468.68	4.66	25.23	Product quality/preparation
Product Deposit	9	95.12	49.69	94.03	848.25	5.58	47.68	Product quality/preparation
Liquid Product Physical Issue	14	21.57	12.81	21.21	277.46	3.65	12.51	Product quality/preparation
Product Physical Issue	5	5.52	2.38	5.49	20.16	1.24	2.37	Product quality/preparation
Product Colour Issue	15	51.54	31.05	50.59	741.39	4.91	30.00	Product quality/preparation

PTs are grouped and ordered according to interpretive relevance rather than the magnitude of the disproportionality estimates. Disproportionality estimates based on small report counts may be unstable and should not be interpreted as measures of incidence or clinical risk magnitude.

IC025 = lower 95% confidence bound of the information component, OE025 = lower 95% confidence bound of the observed-to-expected ratio, PRR = proportional reporting ratio, PT = Preferred Term, ROR = reporting odds ratio, ROR025 = lower 95% confidence bound of the reporting odds ratio.

### 3.4. Time-to-onset

Time-to-onset could be calculated for only 139 of the 823 reports, corresponding to approximately 16.9% of the dataset. Therefore, this analysis was subject to missing date fields and potential selection bias. After outlier removal, the median time-to-onset was 12 days (IQR, 0–57). Among reports with valid date information, 62 events occurred within 0 to 7 days, 29 within 8 to 30 days, 20 within 31 to 90 days, and 28 after more than 90 days (Fig. 2).

**Figure 2.**
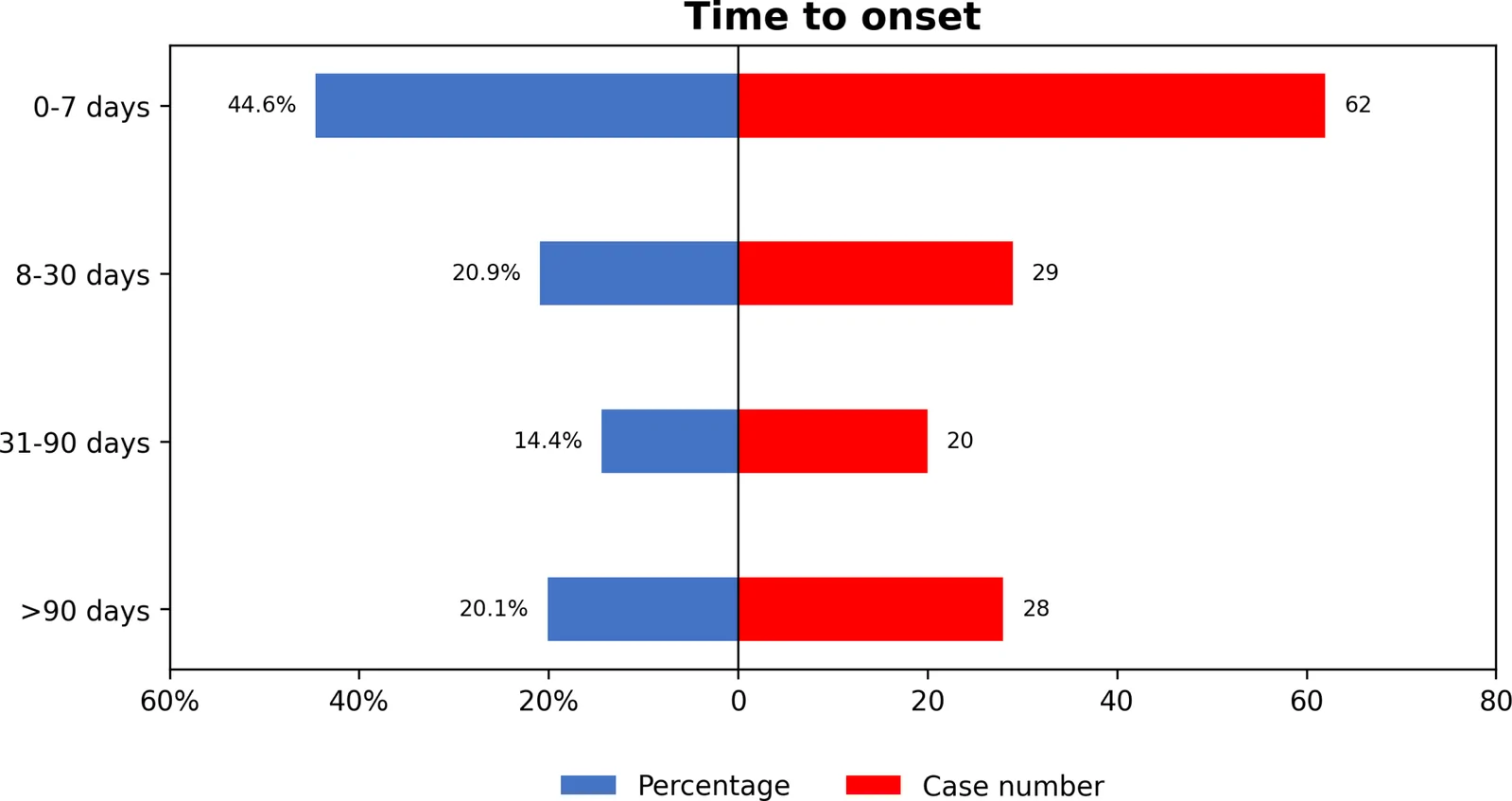
Time to onset of reported adverse events among evaluable reports. Percentages were calculated among evaluable reports with valid event and therapy start dates after exclusion of implausible outliers (n = 139), not among all 823 PS reports. PS = primary suspect.

### 3.5. Sensitivity analyses

Recalculation of the PS analysis reproduced the original results exactly, including 823 target reports and 46 robust PT signals. The all-role analysis included 1137 ensifentrine reports and retained 41 of the 46 primary signals (89.1%; Spearman ρ = 0.952). The single-suspect analysis included 805 target reports and retained 45 signals (97.8%; ρ = 0.991). After excluding Product issues reports, 769 target reports retained 38 signals (82.6%; ρ = 0.995).

The COPD-restricted analysis included 391 target and 16,396 reference reports and retained 22 of the 46 primary signals (47.8%; ρ = 0.769). Bronchospasm, atrial fibrillation, depression, and suicidal ideation remained robust, whereas dyspnea, paradoxical bronchospasm, and chest pain did not meet all 4 criteria. The healthcare-professional analysis included 330 target reports and retained 17 signals (37.0%; ρ = 0.883). Respiratory, thoracic and mediastinal disorders remained the only robust SOC in all analyses except the COPD-restricted analysis, in which Psychiatric disorders was the only robust SOC (Tables S1–S3, Supplemental Digital Content 1).

## 4. Discussion

This study is a report-level disproportionality analysis of early post-marketing FAERS/AEMS data reported with ensifentrine, linking PT-level findings to the MedDRA primary SOC level. The main finding was the predominance of respiratory-related reporting patterns among the 823 reports in which ensifentrine was coded as the PS drug. In the MedDRA primary SOC analysis, the only SOC meeting all 4 predefined signal criteria was “Respiratory, thoracic and mediastinal disorders.” At the PT level, respiratory terms such as dyspnea, bronchospasm, paradoxical bronchospasm, productive cough, sputum increased, sputum discolored, throat tightness, and hemoptysis were prominent.

Disproportionality analyses are useful for identifying reporting imbalances and rare potential safety signals in spontaneous-reporting databases; however, they remain exploratory, cannot quantify clinical risk, and require careful interpretation, particularly in the presence of polypharmacy and potential drug–drug interactions.^[21,22]^

The rapid accumulation of reports after ensifentrine approval may partly reflect a Weber-like reporting pattern. The classical Weber effect describes an increase in adverse event reporting after market entry, often followed by a peak during the early post-marketing years and a subsequent decline.^[23]^ However, the present study covered only the first 7 quarters after approval and, therefore, could not demonstrate the complete temporal pattern. The early increase may also reflect expanding use, heightened awareness among patients and healthcare professionals, or stimulated reporting rather than a true increase in adverse event risk. Without an exposure denominator, changes in report counts cannot be separated from changes in the number of patients receiving ensifentrine.

Notoriety bias should also be considered when interpreting these findings. Publicity, regulatory communications, labeling changes, or heightened awareness during the early post-marketing period may selectively increase the likelihood that particular adverse events are recognized and reported. Such attention may increase report counts and disproportionality estimates without indicating a corresponding increase in event incidence, although evidence suggests that this effect is not uniform across all drugs or safety communications.^[24]^ Because the timing and intensity of media coverage, FDA communications, and labeling changes were not evaluated in the present study, the potential contribution of notoriety bias could not be quantified or excluded.

The predominance of respiratory signals is biologically and clinically plausible. Ensifentrine is an inhaled PDE3/PDE4 inhibitor with bronchodilatory and anti-inflammatory activity and is used as maintenance therapy for COPD.^[25,26]^ The phase III ENHANCE trials and a pooled post hoc analysis in patients receiving long-acting bronchodilators demonstrated improvements in lung function and evaluated ensifentrine in a clinically relevant COPD treatment context.^[5,6]^ Therefore, the appearance of terms such as dyspnea, COPD, cough, sputum, or bronchospasm in FAERS/AEMS reports may reflect the symptom burden of the underlying disease, exacerbation episodes, the reason for treatment initiation, or the reporter’s clinical interpretation rather than a direct drug effect.^[26]^ This is particularly important for the PTs dyspnea and COPD, which should be regarded as disease-confounded terms and should not be presented as causal adverse reactions in the main safety message. Considering potential reporting errors in spontaneous reporting systems, ROR/PRR-based signals should be viewed as exploratory findings that require clinical and biological contextualization; they do not, on their own, provide evidence of causality, incidence, or patient-level risk.^[8,11,12]^

Confounding by indication is particularly relevant in this analysis because ensifentrine is prescribed for COPD, and several detected PTs are also features of the disease. Dyspnea, cough, increased sputum production, and hemoptysis may occur during the natural course of COPD or an exacerbation, while atrial fibrillation and depression are common comorbidities in this population.^[26–28]^ Reports submitted after treatment initiation may therefore attribute these events to ensifentrine even when they reflect baseline disease severity, an exacerbation, or a preexisting condition. Because FAERS/AEMS reports did not consistently provide COPD severity, baseline symptoms, exacerbation status, or complete comorbidity data, these alternative explanations could not be separated from a treatment-related effect. These PTs should therefore be interpreted cautiously and require confirmation in comparative studies with better control of the underlying indication.

In contrast, bronchospasm and especially paradoxical bronchospasm are findings that warrant clinical attention. In this analysis, paradoxical bronchospasm appeared in a small number of reports but showed very high disproportionality estimates. Such high values should be interpreted cautiously because rare events with small cell counts can markedly inflate ROR and related measures.

Nevertheless, the ensifentrine prescribing information explicitly describes the possibility of paradoxical bronchospasm after inhalation and recommends treatment with a short-acting inhaled bronchodilator, discontinuation of ensifentrine, and initiation of alternative therapy if this occurs.^[29]^ Therefore, although it does not establish causality, the paradoxical bronchospasm finding is consistent with an existing label warning and may be considered a clinically prioritizable reporting signal. Its true clinical relevance should be assessed in data sources with an exposure denominator, particularly in new-user cohort studies adjusted for COPD severity, concomitant inhaler use, and exacerbation history.

Extremely large disproportionality estimates should not be interpreted as evidence of a correspondingly large clinical risk. In this analysis, paradoxical bronchospasm (n = 12; ROR = 1149.45), sputum increased (n = 8; ROR = 74.33), and product deposit (n = 9; ROR = 95.12) were based on small exposed-event counts. When the expected background count is very low, the addition or removal of only 1 or 2 reports can substantially change the ROR and produce sparse-cell inflation.^[30]^ The Haldane–Anscombe correction prevents infinite estimates but does not eliminate this instability. These PTs should therefore be interpreted according to their clinical relevance, biological plausibility, consistency with labeling information, and stability across sensitivity analyses rather than the magnitude of the point estimate alone. Product deposit should additionally be viewed as a product-quality report rather than a patient-level adverse event.

Cardiovascular PT-level findings represent a separate area of clinical attention. Terms such as chest discomfort, chest pain, blood pressure increased, atrial fibrillation, and hypertensive crisis met the PT-level signal criteria; however, “Cardiac disorders” did not meet the robust signal criteria in the MedDRA SOC-level analysis. This suggests that PT-level analyses may capture narrower and more specific reporting patterns, whereas SOC-level analyses may dilute individual events within broader organ-system categories.

At the same time, there is a substantial possibility of confounding, given the high burden of cardiovascular comorbidities in patients with COPD, the older age profile of the reported population, concomitant bronchodilator use, and the clinical overlap between cardiac and pulmonary causes of dyspnea or chest symptoms. Therefore, cardiovascular PTs should be reported as potential signals requiring clinical attention, but they should not be interpreted as evidence of an ensifentrine-specific increase in cardiovascular risk.

Psychiatric and neuropsychiatric PT-level findings should be interpreted with similar caution. Depression, suicidal ideation, hallucination, depressed mood, and abnormal dreams were notable at the PT level; however, “Psychiatric disorders” did not fully meet the robust signal criteria at the SOC level. The prescribing information for ensifentrine includes warnings regarding psychiatric adverse reactions, including psychiatric events involving suicidality.^[29]^ Therefore, the PT-level psychiatric findings may be considered clinically relevant reporting signals that are broadly consistent with the label information. Nevertheless, psychiatric comorbidities such as depression and anxiety are common in patients with COPD.^[27,28]^ Accordingly, these findings should be presented not as evidence of drug-induced psychiatric adverse reactions, but as reporting patterns that support clinical awareness and justify further observational research.

From a biological perspective, the cardiovascular and psychiatric findings have possible, although unconfirmed, pharmacological explanations. PDE3 regulates cyclic adenosine monophosphate signaling in cardiac myocytes and vascular smooth muscle; consequently, systemic PDE3 inhibition may influence myocardial contractility, heart rate, and susceptibility to arrhythmias.^[31]^ These mechanisms provide a theoretical context for the cardiovascular PTs observed in this study. However, ensifentrine is administered by inhalation, and the absence of a robust cardiac SOC-level signal, together with the high cardiovascular comorbidity burden of COPD, argues against attributing these reports directly to the drug. Because patients with COPD frequently receive other bronchodilators with potential cardiovascular effects, the observed cardiovascular PTs should also be considered in the broader context of inhaled COPD pharmacotherapy. Evidence regarding the cardiovascular safety of established inhaled COPD therapies is mixed. A systematic review and meta-analysis found no significant increase in cardiovascular events with long-acting muscarinic antagonist (LAMA) therapy compared with placebo,^[32]^ whereas another meta-analysis reported a higher risk of major adverse cardiovascular events with LAMA/long-acting β2-agonist (LABA) or triple therapy than with ICS/LABA, particularly among patients with a higher baseline cardiovascular risk.^[33]^ Accordingly, the atrial fibrillation and hypertension PTs observed in the present analysis may not be unique to ensifentrine and should be interpreted in the context of background cardiovascular disease, concomitant bronchodilator use, and comparator selection.

Systemic PDE4 inhibition also provides a possible class-based context for psychiatric events. The prescribing information for oral roflumilast describes increased psychiatric adverse reactions, including insomnia, anxiety, depression, and suicidality.^[34]^ A recent FAERS pharmacovigilance study of roflumilast similarly identified psychiatric reporting signals, including insomnia and suicidal ideation.^[35]^ Nevertheless, ensifentrine differs from roflumilast in its route of administration, systemic exposure, and dual PDE3/PDE4 profile. Therefore, this class-based comparison supports clinical vigilance but does not establish a shared mechanism, causal relationship, or identical safety profile.

The death reports were heterogeneous and did not cluster within a single clinical category. Half contained respiratory PTs, whereas infection and cardiac terms occurred in 2 and 1 reports, respectively. No report contained an explicit COPD exacerbation PT, although the absence of this term does not exclude unrecorded disease worsening. Psychiatric terms were present in 4 reports, including 3 reports in which completed suicide was the sole PT. Because FAERS/AEMS does not provide adjudicated causes of death or sufficiently detailed case narratives, these patterns cannot establish a causal sequence, mortality incidence, or an ensifentrine-attributable mortality risk. They support continued surveillance and detailed case-level review.

An important methodological feature of this study was the separation of product quality, preparation, effectiveness, and medication-use terms from clinical adverse events. PTs such as product color issue, liquid product physical issue, product preparation issue, product solubility abnormal, product deposit, drug effect less than expected, intentional underdose, and absence of immediate treatment response may show high disproportionality estimates; however, they do not necessarily represent patient-level clinical adverse reactions.

FAERS/AEMS is a spontaneous reporting system that may include not only adverse event reports but also medication errors and product quality complaints. Therefore, separating these terms from the clinical adverse event table was intended to improve the interpretability of the findings.

The relatively frequent reporting of product quality- or preparation-related PTs may suggest the need for pharmacovigilance attention to patient education, device or ampule handling, storage conditions, product appearance, and treatment preparation processes, particularly for newly introduced nebulized or inhaled products. However, these findings should not be equated with clinical safety risks and should instead be interpreted as quality- or use-related reporting signals.

Time-to-onset findings were based on only 139 of the 823 PS reports (16.9%) and should be interpreted cautiously. The availability of treatment-start and event dates may not have been random, as reports with complete dates could differ from those with missing dates in seriousness, reporter type, or clinical follow-up. This informative missingness limits the generalizability of the findings. Reporting delay may also have affected case inclusion because adverse events can be submitted weeks or months after they occur, and reports submitted after the end of the extraction period would not have been captured. Potential left truncation should also be considered because treatment history or events occurring before a report entered FAERS/AEMS may be incompletely observed. In addition, the recorded therapy start date may not represent the patient’s true first exposure. The median time-to-onset of 12 days therefore describes only the selected subset with usable dates and should not be interpreted as a population risk window or evidence of a causal latency.

The findings of this study should not be directly compared with clinical trial data, because phase III trials evaluate adverse event frequencies and efficacy outcomes under controlled conditions, whereas FAERS/AEMS contains voluntary or mandatory real-world reports that are incomplete and subject to reporting selection. Nevertheless, some PT-level findings become clinically meaningful when interpreted alongside the prescribing information and clinical trial context. For example, the terms back pain and blood pressure increased/hypertension are consistent with clinical events listed among common adverse reactions in the prescribing information. In contrast, terms such as dyspnea and COPD may be strongly confounded by disease activity or by the treatment indication itself.

Previous FAERS/AEMS studies suggest that several of the reporting patterns identified in this analysis may not be specific to ensifentrine. In a large pharmacovigilance analysis of tiotropium, respiratory disorders met all 4 signal criteria, and dyspnea and cough were among the most frequently reported PTs.^[36]^ A separate study examining 4 LAMA/LABA combinations – formoterol/glycopyrronium, indacaterol/glycopyrronium, vilanterol/umeclidinium, and olodaterol/tiotropium – also found that dyspnea and cough were commonly reported across these treatments.^[37]^ Respiratory terms are therefore a recurring feature of FAERS/AEMS analyses involving inhaled COPD therapies. They may reflect the underlying disease, COPD exacerbations, inadequate symptom control, inhalation-related effects, or adverse drug reactions. This overlap makes it difficult to determine whether the respiratory signals observed for ensifentrine represent a drug-specific safety pattern.

The cardiovascular and psychiatric findings can also be viewed in the context of previous pharmacovigilance studies. A FAERS/AEMS analysis of inhaled LAMAs found more cardiovascular event reports with glycopyrronium and umeclidinium than with tiotropium, although reporting was lower when these agents were used in LABA-containing combinations.^[38]^ Cardiovascular signals were also identified for some LAMA/LABA combinations, particularly indacaterol/glycopyrronium.^[37]^ In addition, a recent FAERS/AEMS study of roflumilast identified psychiatric signals including insomnia and suicidal ideation.^[35]^ These findings suggest that the respiratory and cardiovascular PTs observed in the present analysis are not confined to ensifentrine and that psychiatric reporting has also been described with another PDE4 inhibitor. However, direct comparison of signal strength across these studies is not appropriate because the analyses differed in treatment route, study period, comparator group, report selection, MedDRA version, and signal-detection criteria.^[20]^

The choice of the entire FAERS/AEMS database as the reference population also affects the interpretation of the findings. Although this approach is useful for broad signal screening, the background database includes patients with indications, demographic characteristics, comorbidities, and reporting patterns that differ substantially from those of patients with COPD. Some of the observed disproportionality may therefore reflect differences between the target and reference populations rather than an effect specific to ensifentrine. Future studies should use COPD-restricted reference populations or active comparators such as roflumilast, LABA/LAMA and ICS/LABA combinations, and nebulized bronchodilators. These comparisons would provide a more clinically relevant background and help determine whether the detected patterns are specific to ensifentrine or shared across COPD treatments.

The sensitivity analyses supported the stability of several principal findings but also showed that some signals depended on the selected population and comparator. The high concordance observed in the all-role, single-suspect, and Product-issues-excluded analyses indicates that the main results were not driven solely by drug-role classification, multiple suspect drugs, or product-quality reports. In contrast, dyspnea and the respiratory SOC were attenuated in the COPD-restricted analysis, supporting the influence of confounding by indication. The lower signal retention in the COPD-restricted and healthcare-professional analyses should also be interpreted in light of their smaller and more selective target populations.

The clinical and research value of this study lies in its systematic and transparent classification of early post-marketing reporting patterns for ensifentrine. The findings indicate that respiratory reports were predominant, that some cardiovascular and psychiatric PTs require clinical attention, and that product quality and medication-use terms should be interpreted separately from patient-level adverse events.

The next research step should be to reassess these findings using comparative sensitivity analyses of all-role and PS-only reports, a COPD-indication subgroup reference set, active comparator designs including similar inhaled COPD therapies, and formal Bayesian shrinkage models. In addition, predefined outcomes such as bronchospasm, cardiovascular events, and psychiatric outcomes should be examined in data sources with an exposure denominator, such as electronic health records or insurance claims databases, using new-user cohort designs. Without such confirmatory studies, the current FAERS/AEMS findings should be regarded as early pharmacovigilance hypotheses rather than definitive evidence for clinical decision-making.

## 5. Conclusion

This study showed that the most prominent reporting pattern in early FAERS/AEMS reports for ensifentrine was related to the respiratory system. At the MedDRA primary SOC level, only Respiratory, thoracic and mediastinal disorders met the robust signal criteria. At the PT level, terms such as bronchospasm, paradoxical bronchospasm, dyspnea, chest pain, depression, and suicidal ideation were notable; however, dyspnea and COPD in particular should be interpreted cautiously because they may overlap with COPD itself. Product quality, effectiveness, and medication-use-related PTs should be evaluated separately from clinical adverse events. These findings do not provide evidence of causality, incidence, or patient-level risk and should be considered early pharmacovigilance signals requiring independent validation. Further validation using COPD-specific comparator groups, all-role sensitivity analyses, and real-world data sources is needed.

## 6. Limitations

The comparator data consisted of all deduplicated FAERS/AEMS reports from the same period; a COPD-restricted or active-comparator reference set was not used. Although appropriate for broad signal screening, this choice may have introduced bias because the background reporting population differs substantially from patients treated for COPD. A separate serious-only disproportionality analysis was not performed because seriousness classifications were not incorporated into the complete background comparator dataset; restricting only ensifentrine reports to serious outcomes without a corresponding serious-report reference population would not provide a comparable disproportionality analysis.

Confounding by indication also remains a major limitation because several detected PTs may reflect COPD, its exacerbations, or associated comorbidities rather than an effect of ensifentrine. The COPD-restricted and healthcare-professional analyses also had smaller target populations, reducing precision and the ability of uncommon events to meet all 4 signal criteria.

The short post-approval observation period may also have been influenced by Weber-like or stimulated reporting, which could not be distinguished from increasing ensifentrine exposure. In addition, the timing and intensity of media coverage, regulatory communications, and labeling changes were not evaluated; therefore, notoriety bias affecting the reporting of particular events could not be quantified or excluded.

A methodological limitation of this study is that formal Bayesian shrinkage methods, such as EBGM/MGPS or full BCPNN, were not applied. Bayesian shrinkage approaches can reduce the instability of disproportionality estimates, particularly for sparse drug-event combinations. In the present analysis, the crude OE metric was retained only as a supportive OE measure and was not interpreted as EBGM, empirical Bayes 5th percentile, MGPS, or formal BCPNN output. Future studies should reassess these findings using formal Bayesian shrinkage models, especially for sparse PTs with very high disproportionality estimates.

The death-report categories were based on co-reported MedDRA PTs rather than adjudicated causes of death and were not mutually exclusive. Time-to-onset was available for only 16.9% of reports and was subject to informative missingness, reporting delay, and potential left truncation.

More broadly, FAERS/AEMS is subject to underreporting and selective reporting. Although deduplication was performed using CASEID, FDA_DT, and PRIMARYID, residual duplicate or follow-up reports may remain when linkage information is incomplete or inconsistent. Important clinical variables including COPD severity, smoking status, comorbidities, baseline psychiatric history, concomitant treatments, dose, treatment adherence, and diagnostic findings were frequently missing or unavailable. Consequently, adjustment for measured and unmeasured confounding was not possible. Finally, because FAERS/AEMS does not provide the number of patients exposed to ensifentrine or person-time at risk, these data cannot be used to estimate incidence, absolute risk, or comparative clinical risk.

## Acknowledgments

The author used ChatGPT (OpenAI) to assist with English language editing, grammar correction, and improvement of writing clarity. The author was fully responsible for the study design, data analysis, interpretation of the results, and the final content of the manuscript. No funds, grants, or other financial support were received for the conduct of this study or the preparation and publication of this manuscript.

## Author contributions

**Data curation**: Fatma Özge Yağbasan.

**Formal analysis:** Fatma Özge Yağbasan.

**Methodology:** Fatma Özge Yağbasan.

**Validation:** Fatma Özge Yağbasan.

**Visualization:** Fatma Özge Yağbasan.

**Writing – original draft**: Fatma Özge Yağbasan.

**Writing – review & editing:** Fatma Özge Yağbasan.








