# Supplementary material for: Post-marketing safety of ensifentrine in COPD: A retrospective pharmacovigilance study using disproportionality analysis of FAERS/AEMS reports
Source: Medicine (Baltimore). 2026 Sep 25;105(39):e50743. doi: 10.1097/MD.0000000000050743 (PMC13619262; doi:10.1097/MD.0000000000050743)
Supplement: Supplementary file 1 [file medi-105-e50743-s001.docx]

**Supplementary Table S1** Sensitivity-analysis populations and signal counts.

| **Scenario** | **Definition** | **Target reports, n** | **Reference reports, n** | **Unique target PTs, n** | **Robust PT signals, n** | **Robust SOC signals, n** | **Robust SOC name(s)** |
| --- | --- | --- | --- | --- | --- | --- | --- |
| Primary PS | Primary-suspect baseline; reproduces the main analysis | 823 | 2,499,888 | 416 | 46 | 1 | Respiratory, thoracic and mediastinal disorders |
| All roles | Ensifentrine recorded in any drug role; all such reports removed from reference | 1,137 | 2,499,574 | 662 | 63 | 1 | Respiratory, thoracic and mediastinal disorders |
| Single suspect | Exactly one unique PS/SS drug in both target and reference reports | 805 | 2,066,636 | 407 | 52 | 1 | Respiratory, thoracic and mediastinal disorders |
| COPD indication | COPD recorded in INDI for both target and reference reports | 391 | 16,396 | 336 | 31 | 1 | Psychiatric disorders |
| Healthcare professional | Reporter occupation MD, PH, OT, or HP in both target and reference reports | 330 | 1,184,948 | 181 | 19 | 1 | Respiratory, thoracic and mediastinal disorders |
| Exclude product issues | Reports containing a MedDRA primary SOC of Product issues excluded from both populations | 769 | 2,350,897 | 381 | 40 | 1 | Respiratory, thoracic and mediastinal disorders |

**Note:** Robust signal counts correspond to the prespecified sensitivity-analysis definitions shown in the table. COPD, chronic obstructive pulmonary disease; HP, health professional; INDI, indication table; MD, physician; MedDRA, Medical Dictionary for Regulatory Activities; OT, other health professional; PH, pharmacist; PS, primary suspect; PT, Preferred Term; SOC, System Organ Class; SS, secondary suspect.
