# Supplementary material for: Post-marketing safety of ensifentrine in COPD: A retrospective pharmacovigilance study using disproportionality analysis of FAERS/AEMS reports
Source: Medicine (Baltimore). 2026 Sep 25;105(39):e50743. doi: 10.1097/MD.0000000000050743 (PMC13619262; doi:10.1097/MD.0000000000050743)
Supplement: Supplementary file 2 [file medi-105-e50743-s002.docx]

**Supplementary Table S4** Co-reported MedDRA Preferred Term categories among reports coded with death as an outcome

| Co-reported category | Reports, n | Percentage |
| --- | --- | --- |
| At least one respiratory PT | 5 | 50.0% |
| At least one infection PT | 2 | 20.0% |
| At least one cardiac or vascular PT | 1 | 10.0% |
| Explicit COPD/exacerbation PT | 0 | 0.0% |
| At least one psychiatric PT | 4 | 40.0% |
| Completed suicide as the sole PT | 3 | 30.0% |
| Metastatic lung cancer PT | 1 | 10.0% |

The denominator was the 10 unique primary-suspect ensifentrine reports coded with death in the OUTC table. Categories were not mutually exclusive, and each report was counted once within each category. The listed PTs were co-reported MedDRA terms and were not adjudicated causes of death. COPD, chronic obstructive pulmonary disease; MedDRA, Medical Dictionary for Regulatory Activities; OUTC, outcome; PT, Preferred Term.
