# Supplementary material for: Post-marketing safety of ensifentrine in COPD: A retrospective pharmacovigilance study using disproportionality analysis of FAERS/AEMS reports
Source: Medicine (Baltimore). 2026 Sep 25;105(39):e50743. doi: 10.1097/MD.0000000000050743 (PMC13619262; doi:10.1097/MD.0000000000050743)
Supplement: Supplementary file 3 [file medi-105-e50743-s003.docx]

**Supplementary Table S2** Stability of selected clinical PT signals across sensitivity analyses.

| **Scenario** | **PT** | **Target PT reports, n** | **ROR** | **ROR025** | **PRR** | **IC025** | **OE025** | **Robust signal** |
| --- | --- | --- | --- | --- | --- | --- | --- | --- |
| All roles | Atrial Fibrillation | 25 | 5.51 | 3.72 | 5.41 | 1.87 | 3.64 | Yes |
| COPD indication | Atrial Fibrillation | 16 | 5.79 | 3.43 | 5.59 | 1.59 | 3.00 | Yes |
| Exclude product issues | Atrial Fibrillation | 20 | 6.33 | 4.08 | 6.19 | 1.99 | 3.98 | Yes |
| Healthcare professional | Atrial Fibrillation | 2 | 1.75 | 0.50 | 1.74 | -0.99 | 0.50 | No |
| Primary PS | Atrial Fibrillation | 20 | 6.13 | 3.95 | 6.00 | 1.95 | 3.86 | Yes |
| Single suspect | Atrial Fibrillation | 20 | 7.40 | 4.77 | 7.24 | 2.22 | 4.66 | Yes |
| All roles | Bronchospasm | 26 | 46.26 | 31.35 | 45.21 | 4.91 | 30.04 | Yes |
| COPD indication | Bronchospasm | 5 | 9.14 | 3.62 | 9.02 | 1.61 | 3.05 | Yes |
| Exclude product issues | Bronchospasm | 20 | 51.56 | 33.13 | 50.21 | 4.99 | 31.76 | Yes |
| Healthcare professional | Bronchospasm | 16 | 73.13 | 44.37 | 69.53 | 5.37 | 41.43 | Yes |
| Primary PS | Bronchospasm | 20 | 49.28 | 31.68 | 48.08 | 4.93 | 30.45 | Yes |
| Single suspect | Bronchospasm | 19 | 58.43 | 37.10 | 57.04 | 5.15 | 35.46 | Yes |
| All roles | Bronchospasm Paradoxical | 12 | 828.67 | 431.63 | 819.58 | 8.32 | 318.71 | Yes |
| COPD indication | Bronchospasm Paradoxical | 2 | 11.07 | 2.74 | 11.01 | 1.19 | 2.28 | No |
| Exclude product issues | Bronchospasm Paradoxical | 12 | 1158.00 | 602.41 | 1139.22 | 8.79 | 442.38 | Yes |
| Healthcare professional | Bronchospasm Paradoxical | 11 | 1983.72 | 961.34 | 1914.83 | 9.30 | 632.35 | Yes |
| Primary PS | Bronchospasm Paradoxical | 12 | 1149.45 | 598.12 | 1132.03 | 8.78 | 439.71 | Yes |
| Single suspect | Bronchospasm Paradoxical | 12 | 1669.51 | 817.88 | 1643.63 | 9.02 | 519.23 | Yes |
| All roles | Chest Pain | 32 | 4.06 | 2.86 | 3.97 | 1.48 | 2.80 | Yes |
| COPD indication | Chest Pain | 9 | 2.44 | 1.26 | 2.40 | 0.26 | 1.20 | No |
| Exclude product issues | Chest Pain | 22 | 4.09 | 2.69 | 4.00 | 1.39 | 2.63 | Yes |
| Healthcare professional | Chest Pain | 13 | 6.14 | 3.56 | 5.93 | 1.78 | 3.44 | Yes |
| Primary PS | Chest Pain | 23 | 4.05 | 2.69 | 3.96 | 1.39 | 2.63 | Yes |
| Single suspect | Chest Pain | 23 | 4.94 | 3.28 | 4.83 | 1.68 | 3.20 | Yes |
| All roles | Depression | 34 | 3.98 | 2.83 | 3.89 | 1.47 | 2.77 | Yes |
| COPD indication | Depression | 15 | 6.74 | 3.91 | 6.52 | 1.75 | 3.36 | Yes |
| Exclude product issues | Depression | 27 | 4.89 | 3.34 | 4.75 | 1.70 | 3.24 | Yes |
| Healthcare professional | Depression | 8 | 4.35 | 2.20 | 4.27 | 1.11 | 2.16 | Yes |
| Primary PS | Depression | 28 | 4.56 | 3.14 | 4.43 | 1.61 | 3.05 | Yes |
| Single suspect | Depression | 25 | 4.84 | 3.26 | 4.72 | 1.67 | 3.18 | Yes |
| All roles | Dyspnoea | 254 | 10.78 | 9.37 | 8.59 | 2.90 | 7.47 | Yes |
| COPD indication | Dyspnoea | 101 | 1.34 | 1.06 | 1.25 | -0.01 | 1.00 | No |
| Exclude product issues | Dyspnoea | 163 | 10.42 | 8.76 | 8.42 | 2.83 | 7.09 | Yes |
| Healthcare professional | Dyspnoea | 52 | 7.39 | 5.50 | 6.38 | 2.25 | 4.76 | Yes |
| Primary PS | Dyspnoea | 174 | 10.04 | 8.49 | 8.13 | 2.78 | 6.88 | Yes |
| Single suspect | Dyspnoea | 171 | 11.16 | 9.43 | 9.00 | 2.93 | 7.61 | Yes |
| All roles | Suicidal Ideation | 22 | 5.42 | 3.57 | 5.33 | 1.81 | 3.51 | Yes |
| COPD indication | Suicidal Ideation | 14 | 50.35 | 23.47 | 48.52 | 3.59 | 12.06 | Yes |
| Exclude product issues | Suicidal Ideation | 22 | 7.87 | 5.17 | 7.67 | 2.33 | 5.03 | Yes |
| Healthcare professional | Suicidal Ideation | 14 | 13.99 | 8.25 | 13.42 | 2.98 | 7.90 | Yes |
| Primary PS | Suicidal Ideation | 22 | 7.55 | 4.96 | 7.37 | 2.27 | 4.83 | Yes |
| Single suspect | Suicidal Ideation | 21 | 8.11 | 5.28 | 7.92 | 2.36 | 5.14 | Yes |

**Note:** Values are displayed to two decimal places. “Robust signal” indicates whether all four prespecified signal criteria were simultaneously met in the corresponding scenario. COPD, chronic obstructive pulmonary disease; IC025, lower 95% confidence bound of the Information Component; OE025, lower 95% confidence bound of the observed-to-expected ratio; PRR, Proportional Reporting Ratio; PS, primary suspect; PT, Preferred Term; ROR, Reporting Odds Ratio; ROR025, lower 95% confidence bound of the Reporting Odds Ratio.
