# Supplementary material for: Post-marketing safety of ensifentrine in COPD: A retrospective pharmacovigilance study using disproportionality analysis of FAERS/AEMS reports
Source: Medicine (Baltimore). 2026 Sep 25;105(39):e50743. doi: 10.1097/MD.0000000000050743 (PMC13619262; doi:10.1097/MD.0000000000050743)
Supplement: Supplementary file 4 [file medi-105-e50743-s004.docx]

**Supplementary Table S3** PT-level concordance with the primary analysis.

| **Scenario** | **Baseline robust PTs, n** | **Scenario robust PTs, n** | **Overlapping robust PTs, n** | **Baseline signal retention, %** | **Jaccard index** | **Spearman correlation (log ROR)** | **Common PTs for correlation, n** |
| --- | --- | --- | --- | --- | --- | --- | --- |
| Primary PS | 46 | 46 | 46 | 100.00 | 1.000 | 1.000 | 416 |
| All roles | 46 | 63 | 41 | 89.13 | 0.603 | 0.952 | 416 |
| Single suspect | 46 | 52 | 45 | 97.83 | 0.849 | 0.991 | 407 |
| COPD indication | 46 | 31 | 22 | 47.83 | 0.400 | 0.769 | 336 |
| Healthcare professional | 46 | 19 | 17 | 36.96 | 0.354 | 0.883 | 181 |
| Exclude product issues | 46 | 40 | 38 | 82.61 | 0.792 | 0.995 | 381 |

**Note:** Baseline signal retention is presented as a percentage; Jaccard and Spearman correlation values are displayed to three decimal places. COPD, chronic obstructive pulmonary disease; PS, primary suspect; PT, Preferred Term; ROR, Reporting Odds Ratio.
